# Spermatic Cord Anesthesia Block: An Old Technique Re-imaged

**DOI:** 10.5811/westjem.2016.8.31017

**Published:** 2016-09-13

**Authors:** Jeffrey Gordon, Robert P. Rifenburg

**Affiliations:** Presence Resurrection Medical Center, Department of Emergency Medicine, Chicago, Illinois

## Abstract

Spermatic cord anesthesia block (SCAB) is a useful technique for providing anesthesia to males with scrotal pain. This technique has been described and published in the urology and anesthesia literature for more than 40 years. Initially described as a blind injection, anesthesia of the spermatic cord provides pain control to the scrotal contents. The technique can easily be performed under ultrasound guidance by emergency physicians and should be considered a useful option when seeking to provide pain relief to male patients with scrotal pain.

## CASE REPORT

A 37-year-old male presented to the emergency department (ED) with a one-week history of left-sided scrotal pain. He denied previous trauma, associated fever, abdominal pain, hematuria, or any past genitourinary-related medical history. He was previously evaluated for a similar complaint five days earlier at an outside institution. At that time, his physical exam was unremarkable and he was treated for presumed epididymitis with oral antibiotics. However, his symptoms had not improved with this treatment. Upon arrival to our ED, his abdomen was soft with no guarding or palpable mass. He was a circumcised male with normal external genitalia without notable abnormality to the penis or scrotum. His testicles were bilaterally descended in normal anatomic position and there was no inguinal lymphadenopathy or evidence of scrotal cellulitis. His cremasteric reflex was intact. He was however, tender to palpation along the left testicle/epididymis. A radiology department ultrasound was performed, which showed mildly increased vascular flow to the left testes. His pain had not improved and he was subsequently offered a spermatic cord anesthesia block (SCAB) for pain management.

## DISCUSSION

The SCAB technique has been described multiple times previously in the literature.^1^,^2^ As early as 1960, Earle published an article in the *American Journal of Surgery* discussing local anesthesia options for inguinal herniorrhaphy, which described the technique without naming it as such.^3^ The spermatic cord (SC) is a distinct structure in males containing the vas deferens, which exits the abdomen and extends from the deep inguinal ring down to each testicle. The cord is covered by the tunica vaginalis, an extension of the peritoneum. Along with the vas deferens, contained within the SC are the testicular and cremasteric arteries, lymphatic vessels, the pampiniform plexus of veins, and two key nerves – the genital branch of the genitofemoral nerve and the ilioinguinal nerve. The ilioinguinal nerve arises off the 12^th^ thoracic and first lumbar nerve. The genitofemoral nerve arises off the first and second lumbar nerves.^2^ Combined, these nerves provide enervation to the cremasteric muscles and sensation to the intrascrotal contents.^2^ A correctly performed SCAB provides anesthesia to the scrotal contents without providing scrotal skin anesthesia.^10^

Most previously published case series describe a blind technique whereby the SC is identified by manual palpation.^10^ A needle is inserted to deliver anesthetic medication based on tactile location of the cord. The landmark for this procedure is classically described as being a point 1 cm below and 1 cm medial to the pubic tubercle.^4^ The technique as described by Kaye et al was proposed to facilitate vasovasotomy, hydrocelectomy, spermatocelectomy, and orchiectomy^4^ and has been generally viewed as a successful technique.^2^ Both Kaye and Cassady describe a technique involving three needle passes at slightly different angles to the SC with total deposition of 12–15ml of local anesthetic.^2^,^4^ Subsequent articles have commented on the difficulty in palpating and identifying the pubic tubercle,^9^ especially in patients with protuberant abdomens or large pannus folds. These case reports and studies involving SCAB have primarily been published in the urology and anesthesia literature.

The SCAB technique has been proposed as a cost-savings option to facilitate various surgical procedures including outpatient orchiectomy^5^ and vasectomy reversal.^6^ It has also been proposed for treatment of SC torsion prior to manual reduction.^7^,^8^ Kiesling et al report a case series of 15/16 successful detorsions following SCAB.^8^ Some reported advantages to this technique include the lack of need for general anesthesia and its attendant potential complications.^4^ Additionally, patients require less post-operative pain control as the block serves as its own anesthetic resulting in an overall cost savings for the technique compared with general anesthesia.^5^ Reported complications of the blind injection technique include vascular injury to the testicular artery^6^ or possible intra-arterial injection and/or damage to the deferent ducts.^9^ As the availability of ultrasound (US) for emergency physicians continues to increase, SCAB under ultrasound (US) guidance is a simple technique that can provide immediate anesthesia for patients with testicular and scrotal pain.

**Figure 1:**
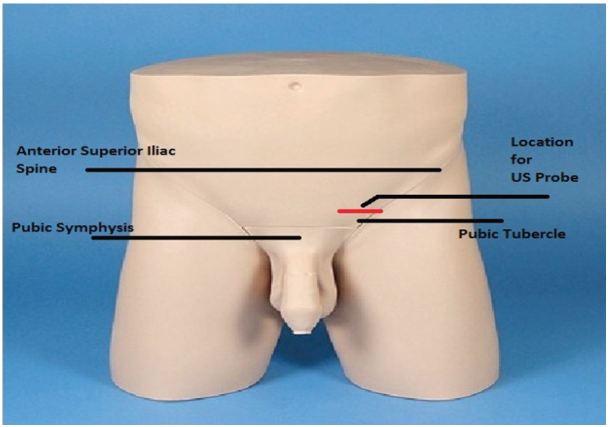
Initial positioning of the ultrasound (US) probe to locate the spermatic cord.

**Figure 2:**
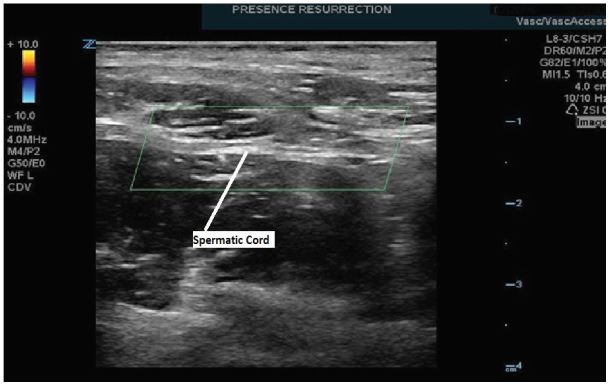
Transverse view of the spermatic cord.

**Figure 3:**
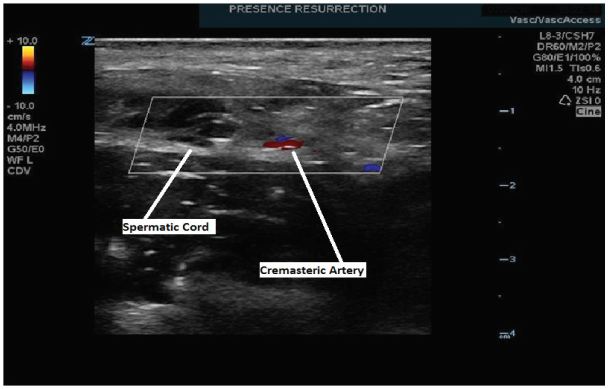
Identification of adjacent vascular structure. Transverse view showing the spermatic cord and adjacent cremasteric artery.

**Figure 4:**
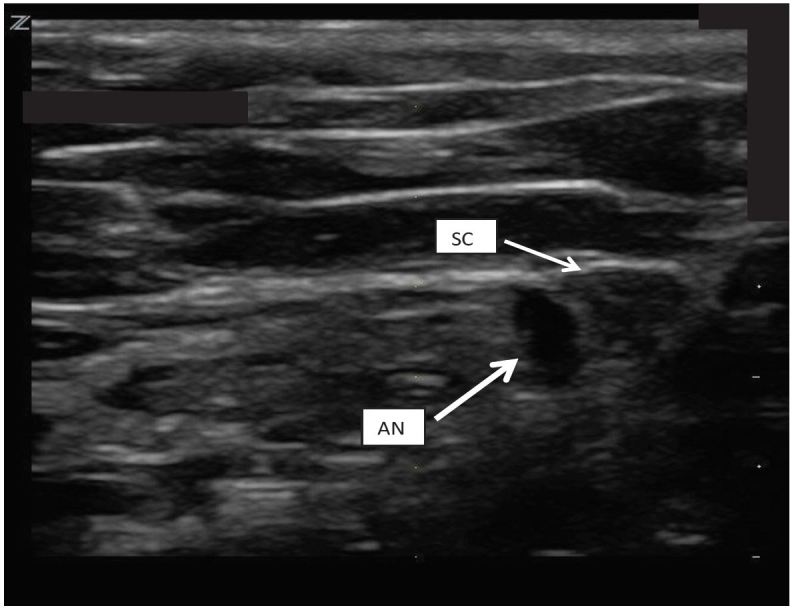
Coronal view. Sonographic anatomy of the spermatic cord (SC) and anesthesia (AN) solution deposited adjacent to the cord.

The SC block performed on our patient was achieved with a multifrequency linear L8-3 probe on our ZONARE Z1 Ultra ultrasound machine. The technique involved first identifying the spermatic cord and cremasteric artery. The probe was positioned between the pubic tubercle and the anterior superior iliac spine on the affected side (Figure 1). Once the SC was identified (Figure 2), 5ml of 1% xylocaine and 5 ml of 0.5% bupivacaine were combined in a single syringe with a #21 gauge 1.5-inch needle. The skin site was prepared and draped and the SC was palpated. The SC location was confirmed by bedside US in both the longitudinal and transverse planes. Under direct US visualization, the needle was positioned in the SC, avoiding the vascular structures (Figure 3). Approximately 8 cc’s of the anesthetic solution was injected in and directly around the SC (Figure 4). The patient reported nearly immediate symptomatic relief without bleeding at the injection site. The patient was monitored for pain relief and was ready for discharge within 15 minutes of nerve block completion. A subsequent follow-up phone call confirmed that our patient did not have any delayed complications nor did he experience a recurrence of his pain.

We present the technique of SCAB under ultrasound guidance. This technique has been described for more than 40 years and has been shown to be an effective adjunct for addressing pain in patients with testicular and/or scrotal complaints. The first step in the management of testicular pain without acute surgical findings remains conservative in nature. Consideration should include the use of scrotal elevation, NSAIDS, and cold compresses. Additionally, US-guided SCAB is a simple effective adjunct. As US availability in the ED is readily accessible, this technique is easily and safely performed by emergency physicians and should be considered a viable option for treating testicular pain in the ED.
